# Genome Sequences of Four Nonhuman/Nonclinical *Salmonella enterica* Serovar Kentucky ST198 Isolates Recovered between 1972 and 1973

**DOI:** 10.1128/genomeA.01699-16

**Published:** 2017-03-16

**Authors:** Seon-Woo Kim, Bradd J. Haley, Dwayne Roberson, Marc Allard, Thomas S. Hammack, Eric W. Brown, Jo Ann S. Van Kessel

**Affiliations:** a Environmental Microbial and Food Safety Laboratory, Beltsville Area Research Center, Agricultural Research Services, United States Department of Agriculture, Beltsville, Maryland, USA; b Division of Microbiology, Office of Regulatory Science, Center for Food Safety and Nutrition, U.S. Food and Drug Administration, College Park, Maryland, USA

## Abstract

*Salmonella enterica* serovar Kentucky is a polyphyletic member of* S. enterica* subclade A1 with multiple sequence types that often colonize the same hosts but in different frequencies on different continents. To evaluate the genomic features involved in *S*. Kentucky host specificity, we sequenced the genomes of four isolates recovered in the 1970s.

## GENOME ANNOUNCEMENT

In North America, *Salmonella enterica* serovar Kentucky is an infrequent human pathogen, but it is frequently isolated from human clinical cases in Europe, North Africa, the Middle East, and Asia (1–7). There is a relatively high prevalence of sequence type 152 (ST152) among *S*. Kentucky isolates in food-producing animals in North America, and an apparently higher prevalence of ST198 than ST152 in human clinical cases and food-producing animals in Europe, Africa, and South Asia (http://mlst.warwick.ac.uk/mlst/dbs/Senterica). Differences in genome content between the two STs may be responsible for the difference in the number of reported *S*. Kentucky infections in Europe and by travelers to ST198 endemic regions than in the United States. To further elucidate the geographic and temporal diversity of *S*. Kentucky and the factors involved in persistence in food-producing animals and virulence in humans, we sequenced the genomes of four ST198 isolates collected from nonhuman/nonclinical sources in the Americas between 1972 and 1973.

Isolates SAL2606 and SAL2608 were recovered from liver meal in Argentina in 1972 and 1973. Isolate SAL2609 was isolated from meat and bone meal in Canada in 1973, and SAL2607 was isolated from raw oysters in 1973. All isolates were susceptible to a panel of 14 antibiotics (amoxicillin/clavulinic acid, ampicillin, azithromycin, cefoxitin, ceftiofur, ceftriaxone, chloramphenicol, ciprofloxacin, gentamicin, nalidixic acid, streptomycin, sulfisoxazole, tetracycline, and trimethoprim-sulfamethoxazole).

To sequence the genomes of these isolates, libraries were constructed using the Nextera XT library prep kit (Illumina, La Jolla, CA), which were then sequenced using a high-output version 2.0 flow cell on a NextSeq 500 platform (Illumina). Sequencing reads were cleaned and trimmed using Deconseq (8) and Trimmomatic (9) and then assembled using SPAdes version 3.8.0 (10). The resulting assemblies ranged between 4.7 Mb and 5.1 Mb, the number of contigs ranged between 55 and 95, and the average read coverage per base ranged between 129× and 236×.

All isolates were confirmed to belong to ST198 (11). Acquired antibiotic resistance genes were not detected among any of the genomes (12). Further, there were no ciprofloxacin-conferring substitutions in the quinolone resistance-determining regions (QRDR) of *gyrA* and *parC*. The multidrug resistance-conferring *Salmonella* genomic island-1 (SGI-1), identified in many ST198 isolates from Africa, Europe, the Middle East, and South Asia, was not detected in any of the genomes. However, an approximately 17-kb island was inserted at the homologous *trmE-yidY* insertion locus in SAL2606, SAL2607, and SAL2609 but not in SAL2608. An IncFIB plasmid replicon was detected in SAL2607, but plasmid replicons were not detected in any of the other genomes. The inclusion of these and other genomes with high-quality metadata will help further elucidate the geophylogeny of *S*. Kentucky ST198.

### Accession number(s).

This genome sequence project has been deposited in DDBJ/ENA/GenBank under the accession numbers MLJQ00000000 (SAL2606), MLJR00000000 (SAL2607), MLJS00000000 (SAL2608), and MLJT00000000 (SAL2609). The versions described in this paper are the first versions, MLJQ00000000.1, MLJR00000000.1, MLJS00000000.1, and MLJT00000000.1, repsectively.
